# Comparison of Changes in Chest Wall Mechanics and Respiratory Timing Between Patient-Controlled Epidural Analgesia and Intravenous Patient-Controlled Analgesia After Laparoscopic Gastrectomy: A Randomized Controlled Trial

**DOI:** 10.7759/cureus.37276

**Published:** 2023-04-08

**Authors:** Masako Asada, Keiko Nobukuni, Jun Yoshino, Naoyuki Fujimura

**Affiliations:** 1 Anesthesiology, Kyushu University Hospital, Fukuoka, JPN; 2 Anesthesiology, Japan Community Healthcare Organization Kyushu Hospital, Kitakyushu, JPN; 3 Anesthesiology, St. Mary’s Hospital, Our Lady of the Snow Social Medical Corporation, Kurume, JPN

**Keywords:** randomized clinical trial, diaphragmatic dysfunction, diaphragmatic function, respiratory timing, chest wall mechanics, impedance plethysmography, laparoscopic gastrectomy, intravenous patient-controlled analgesia, patient-controlled epidural analgesia

## Abstract

Background: Upper abdominal surgery is associated with postoperative diaphragmatic dysfunction. Whether patient-controlled epidural analgesia (PCEA) is superior to intravenous patient-controlled analgesia (IV-PCA) in preventing postoperative diaphragmatic dysfunction is still unclear in laparoscopic gastric surgery.

Methods: Sixteen patients undergoing laparoscopic gastrectomy randomly received either PCEA or IV-PCA. The primary outcomes were the change in chest wall mechanics and respiratory timing, measured by respiratory inductive plethysmography (Respitrace; Ambulatory Monitoring Inc., Ardsley, New York, United States) before and after surgery, and analyzed by a data acquisition system (PowerLab; ADInstruments, Dunedin, New Zealand). Inspiratory time (Ti), expiratory time (Te), total respiratory cycle time (Ttot), proportion of inspiratory time over total respiratory cycle time (Ti/Ttot), respiratory rate (RR), and abdominal contribution to tidal volume (AB/V_T_ [%]) were calculated from the stored data. AB/V_T_, relative volume contribution of diaphragm to tidal breathing, represents an index of diaphragmatic function. Because the diaphragm is the main contributor to tidal volume, decreases in AB/V_T_ indicate diaphragm dysfunction. Changes in outcomes over time between the two groups were analyzed using a linear mixed model, and two-sided *p* values < 0.05 were considered statistically significant. The secondary outcomes were postoperative pain score (visual analog scale (VAS)), bowel function recovery, and hospital stay duration.

Results: Postoperative AB/V_T_ in the IV-PCA group was significantly decreased compared to preoperative levels. AB/V_T_ in the PCEA group was significantly higher than the IV-PCA group on postoperative day (POD) 1. Change in AB/V_T_ over time between the PCEA group and the IV-PCA group differed significantly (*p* = 0.01). A decrease of AB/V_T_ during POD 1 to 3 was observed in the IV-PCA group but not in the PCEA group. As for respiratory timing, there were significant increases in RR with a reduction of Te and Ttot compared to preoperative levels on POD 1 in the PCEA group. There were significant decreases in RR and Ti/Ttot with an increase of Te and Ttot compared to preoperative levels on POD 1 in the IV-PCA group. There was a significant difference in the change of the Ttot over time between the two groups (*p* = 0.046). There were no significant differences in the changes of Te, Ti/Ttot, Ti, and RR over time between the two groups. There was no significant difference in VAS over time at rest and mobilization, recovery of bowel function, and hospital stay between the two groups.

Conclusions: Continuous ropivacaine infusion with PCEA partially attenuated diaphragmatic dysfunction after laparoscopic gastrectomy, while pain relief by continuous intravenous administration of fentanyl could not attenuate diaphragmatic dysfunction. This suggests that PCEA might ameliorate postoperative diaphragmatic dysfunction after laparoscopic gastrectomy.

## Introduction

It is well known that patients undergoing upper abdominal surgery develop a restrictive pattern of ventilation with a significant reduction in vital capacity (VC), forced expiratory volume in one second (FEV_1_), and functional residual capacity (FRC) [1,2]. It is suggested that respiratory muscle dysfunction is responsible for the alterations in pulmonary function after upper abdominal surgery [3,4]. Although the etiology of respiratory muscle dysfunction after upper abdominal surgery is not fully understood, postoperative pain [5] and diaphragmatic dysfunction [6,7] are considered causes of respiratory muscle dysfunction after upper abdominal surgery.

Recently, laparoscopic surgery has been widely performed with the advantages of reduced morbidity with early recovery and decreased postoperative pain. Compared with open abdominal surgery, laparoscopic surgery has been demonstrated to minimize the reduction in postoperative pulmonary function in cholecystectomies [8,9] and colectomies [10]. However, postoperative pulmonary function is significantly reduced after laparoscopic gastric surgery under systemic analgesia with opioids [11].

Epidural analgesia is widely accepted as the gold standard of pain relief after upper abdominal surgery. Epidural analgesia reduces postoperative pulmonary complications after thoracic and abdominal surgery compared with systemic analgesia with opioids [12]. Epidural analgesia improves diaphragmatic dysfunction after upper abdominal surgery [13,14]. However, the role of epidural analgesia after laparoscopic gastric surgery is controversial. Epidural analgesia reduces the need for additional analgesics after laparoscopic gastric surgery, although it might increase the risk of urinary retention [15]. Analgesia with non-steroidal anti-inflammatory drugs and acetaminophen, supplemented with intravenous opioids were used as an enhanced recovery program [16] for laparoscopic gastric surgery. Therefore, it is essential to identify benefits for the patient when using epidural analgesia for laparoscopic surgery to prevent diaphragmatic dysfunction after surgery.

Thus, this study aims to compare the effect of patient-controlled epidural analgesia (PCEA) with background infusion, versus intravenous patient-controlled analgesia (IV-PCA) on diaphragmatic function after laparoscopic gastrectomy. We used respiratory inductive plethysmography to measure diaphragmatic function after surgery.

## Materials and methods

This study was a single-center randomized controlled trial performed from June 2015 to December 2016 at St. Mary’s Hospital, Fukuoka Prefecture, Japan. The study protocol was examined and approved by the Research Review Board at St. Mary’s Hospital on April 14, 2015 (Authorization number: KEN14-0303), and was registered to the University hospital Medical Information Network-Clinical Trial Repository (UMIN-CTR) (UMIN000033021). Written informed consent was obtained from all patients who participated in this study. The study was compliant with the Declaration of Helsinki (as revised in 2013).

We enrolled patients scheduled for laparoscopic gastrectomy, aged 20 years or older, and with the American Society of Anesthesiologists' physical status (ASA-PS) classification of I or II. The exclusion criteria were as follows: (1) patient’s refusal to participate in the study; (2) contraindication to insert an epidural catheter (e.g., coagulopathy, localized infection on the back); (3) patients with an underlying serious disease; and (4) conversion to open abdominal surgery, or discontinuation of surgery due to peritoneal dissemination. All eligible patients received information about both PCEA and IV-PCA as two established postoperative pain control methods. Patients were randomly divided into two groups by sealed envelopes: the PCEA group and IV-PCA group.

Epidural analgesia

In the PCEA group, the epidural catheter was placed without sedation before induction of general anesthesia via a paramedian approach in the lateral decubitus position. After local anesthesia with 1% lidocaine, a Tuohy 17-G epidural needle was inserted at Th7/8, 8/9, or 9/10 intervertebral space and advanced using the loss of resistance technique. Once the epidural space was accessed, the catheter was advanced 4-5 cm into the epidural space in the direction of the cephalad. After a test aspiration was negative, a 3-mL test dose of 1% lidocaine without epinephrine was administered.

General analgesia

Premedication and preoperative multi-modal pain medication were not given to the patients. A standard monitor (i.e., electrocardiogram, indirect blood pressure measurement, and pulse oximeter) was attached to the patients after entering the operating room. General anesthesia was induced with an intravenous injection of fentanyl (1-2 μg/kg) and propofol (1.5-2 mg/kg). Endotracheal intubation was facilitated by the intravenous administration of rocuronium bromide (0.6 mg/kg). Ventilation was controlled to maintain the end-tidal carbon dioxide (CO_2_) at 35-45 mmHg with the fraction of inspiratory oxygen (O_2_) at 40-50%. Positive end-expiratory pressure of 5 cmH_2_O was used. Laparoscopic surgery was performed with intraabdominal placement of the trochar, followed by CO_2_ insufflation of the abdominal cavity to a pressure of 8-10 mmHg during surgery in both groups.

In the PCEA group, anesthesia was maintained with sevoflurane (1.0-2.0%) and an initial bolus injection of 5-10 mL of 0.375% ropivacaine just before surgery, followed by intermittent bolus administration of 4-6 mL of 0.375% ropivacaine via epidural catheter. In the IV-PCA group, anesthesia was maintained with sevoflurane (1.0-2.0%) and remifentanil (0.05-0.50 μg/kg/min). In both groups, intravenous fentanyl and rocuronium were administered as needed. Flurbiprofen (50 mg) and/or acetaminophen (15 mg/kg or 1000 mg) were administered intravenously at the end of surgery in both groups. Sugammadex (2-4 mg/kg) was used to reverse neuromuscular blockage before extubation. In the PCEA group, the level of epidural analgesia was checked by assessing the loss of cold sensation between Th6 and Th12 after emergence and prior to leaving the operating room.

Postoperative analgesia

In the PCEA group, postoperative analgesia was initiated with bolus administration of 4-6 mL of 0.375% ropivacaine at the end of surgery via an epidural catheter. The PCEA device (Coopdech Syrinjector; Daiken Medical, Osaka, Japan) was connected to the patient’s epidural catheter. A standardized analgesic solution of 0.2% ropivacaine and fentanyl 2 μg/kg was used. The quantity of PCEA, namely, background infusion rate (4-6 mL/h), was determined by the anesthesiologist in charge of the patient, with a device-specific bolus dose (3 mL) and lockout intervals (30 minutes).

In the IV-PCA group, the IV-PCA device (CADD-Legacy; Smiths Medical, Inc., Minneapolis, Minnesota, United States) was connected to the patient before the end of surgery. A standardized analgesic solution of fentanyl 10 μg/mL was used. The quantity of IV-PCA was determined by the anesthesiologist in charge of the patient, with reference to standard protocol (i.e., background infusion rate 0.5 μg/kg/h, bolus dose 2 mL, and lockout time intervals 10 minutes).

In both groups, the pump infusion of analgesics was continued until postoperative day (POD) 2. Intravenous flurbiprofen or acetaminophen was used for rescue analgesia when the pain was not well controlled (request for rescue analgesia) irrelevant of PCEA with background infusion or IV-PCA uses.

Outcomes

The primary outcomes of this study were the change in chest wall mechanics and respiratory timing, which was measured by respiratory inductive plethysmography (Respitrace; Ambulatory Monitoring Inc., Ardsley, New York, United States) and analyzed by a data acquisition system (PowerLab; ADInstruments, Dunedin, New Zealand). The respiratory inductive plethysmography device was attached to the patients in the supine position to analyze the change in the chest and abdominal wall on preoperative day 1, PODs 1, 3, 5, and 7. The device consists of two coils sewn into elastic bands connected to an oscillator module. The rib cage band was placed around the chest at nipple level. The abdominal band was placed around the abdomen at the umbilical level. Change in the cross-sectional area of the rib cage and abdominal compartments alters the self-inductance of the wires and the frequency of their oscillators. The rib cage and abdominal signals were summed and recorded electronically to provide a signal equivalent to tidal volume (V_T_). The device was calibrated for each patient in the supine position during quiet breathing. Inspiratory time (Ti), expiratory time (Te), total respiratory cycle time (Ttot), proportion of inspiratory time over total respiratory cycle time (Ti/Ttot), respiratory rate (RR), and abdominal contribution to tidal volume (AB/V_T_ [%]) were calculated from the sampled data. AB/V_T_ represents an index of diaphragmatic function, namely, diaphragmatic contribution to the breathing process [14,17]. Because the diaphragm is the main contributor to tidal volume, decreases in AB/V_T_ indicate diaphragm dysfunction. V_T_ of every breath over periods of five minutes was taken and expressed as means.

The secondary outcomes were postoperative pain scores measured by visual analog scale (VAS) [18], the recovery of bowel function after surgery (day to the first passing of flatus, stool, and day to first soft diet intake), and duration of postoperative hospital stay. Each anesthesiologist evaluated VAS upon leaving the operating room and after that at six and 12 hours after surgery, every morning and evening by each nurse taking charge of the patient. The recovery of bowel function was evaluated and recorded by surgeons and nurses.

Statistical analysis

Data are presented as mean ± standard deviation, least squares mean ± standard error of the least squares mean, median (interquartile range), and number of patients or frequencies. Comparisons between the two groups were performed using unpaired t-test, chi-square test, Mann-Whitney U test, or Fisher’s exact test, as appropriate. Continuous variables with repeated measures (i.e., AB/V_T_, respiratory timing, and VAS) were analyzed using a linear mixed model with patients as a random effect, and group, time, and group-by-time as fixed effects. The group-by-time interaction term evaluates whether the change of the variables over time differs between the two groups. Two-sided *p* values < 0.05 were considered statistically significant in all analyses. All analyses were performed using SAS software package version 9.4 (2013; SAS Institute Inc., Cary, North Carolina, United States).

## Results

Patient characteristics are shown in Table 1. There was no evidence of significant differences between the two groups, except for age and body mass index (BMI). The mean age in the IV-PCA group was significantly higher than in the PCEA group. The mean BMI in the PCEA group was significantly higher than in the IV-PCA group.

**Table 1:** Patient characteristics *p values are based on unpaired t-test or chi square test unless otherwise specified. †Mann-Whitney U test, ‡Fisher's exact test. Abbreviations: ASA-PS, American Society of Anesthesiologists physical status; IQR, interquartile range; IV-PCA, intravenous patient-controlled analgesia; NSAIDs, non-steroidal anti-inflammatory drugs; PCEA, patient-controlled epidural analgesia; SD, standard deviation

Variables	PCEA group (n = 8)	IV-PCA group (n = 8)	*p* value^*^
Pre-operative factors			
Age, mean (SD), years	52 (14)	64 (7)	0.04
Male/female	4 / 4	5 / 3	1.00^‡^
Body mass index, mean (SD), kg/m^2^	26 (4)	21 (3)	0.02
ASA-PS, I / II	6 / 2	3 / 5	0.31^‡^
Operative factors			
Duration, anesthesia, median (IQR), min	392 (303–475)	423 (355–476)	0.67^†^
Duration, surgery, median (IQR), min	260 (202–344)	361 (282–399)	0.14^†^
Total fentanyl dose, mean (SD), μg/kg	5.6 (1.9)	7.0 (1.6)	0.13
Total remifentanil dose, mean (SD), mg	–	5.1 (1.7)	–
Total rocuronium dose, mean (SD), mg/kg	1.6 (0.4)	1.5 (0.5)	0.68
Fluid intake, mean (SD), mL	3120 (147)	2564 (128)	0.31
Blood loss, median (IQR), mL	50 (5–185)	90 (18–213)	0.75^†^
Post-operative factors			
Acetaminophen, median (IQR), per post-operative 5 days	0 (0–0)	0 (0–0)	1.00^†^
NSAIDs, median (IQR), per post-operative 5 days	2.0 (1.5–5.5)	2.5 (1.5–3.5)	0.83^†^

**Figure 1:**
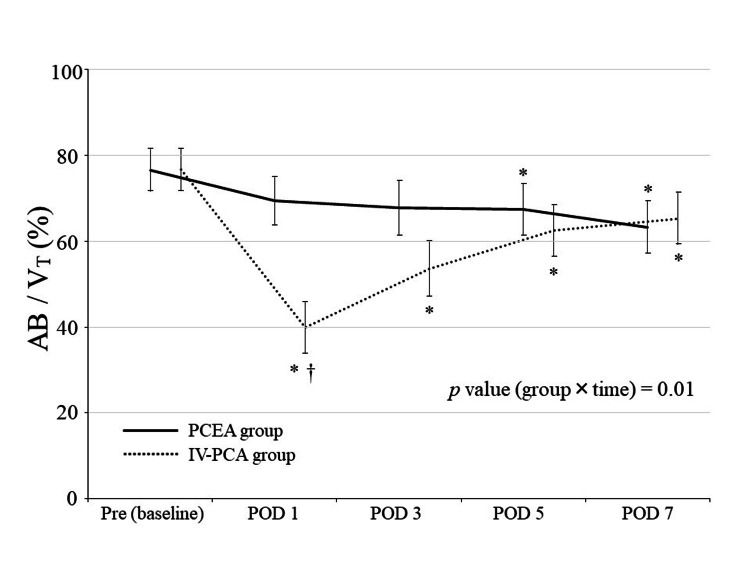
Changes in abdominal contribution to tidal volume after laparoscopic gastrectomy Data are shown as least squares mean ± standard error of the least squares mean. Pre indicates preoperative day 1. *p < 0.05 vs. baseline; †*p* < 0.05 vs. PCEA group. Abbreviations: AB/V_T_, abdominal contribution to tidal volume; IV-PCA, intravenous patient-controlled analgesia; PCEA, patient-controlled epidural analgesia; POD, postoperative day.

Figure 1 shows the changes in AB/V_T_ after laparoscopic gastrectomy. Postoperative AB/V_T_ in the IV-PCA group was significantly decreased compared to preoperative levels. AB/V_T_ in the PCEA group was significantly higher than in the IV-PCA group on POD 1. There was a significant difference in AB/V_T_ over time between the two groups (*p* = 0.01).

Changes in respiratory timing after laparoscopic gastrectomy are shown in Table 2. There were significant differences in Te, Ttot, Ti/Ttot, and RR between the two groups on POD 1. In the PCEA group, there were significant increases in RR with reduced Te and Ttot compared to preoperative levels on POD 1. In the IV-PCA group, there were significant decreases in RR and Ti/Ttot with increase of Te and Ttot compared to preoperative levels on POD 1. There was a significant difference in the change of the Ttot over time between the two groups (*p* = 0.046). There were no significant differences between the two groups in the changes of Te, Ti/Ttot, Ti, and RR over time.

**Table 2:** Changes in respiratory timing after laparoscopic gastrectomy Values are shown as least squares means (standard error). *p < 0.05 vs baseline; †*p* < 0.05 vs PCEA group. Abbreviations: IV-PCA, intravenous patient-controlled analgesia; PCEA, patient-controlled epidural analgesia; POD, postoperative day.

Respiratory timing	Time	PCEA group (n = 8)	IV-PCA group (n = 8)	*p* value (group×time)
Inspiratory time (Ti), second	Pre (baseline)	1.9 (0.3)	2.0 (0.3)	0.17
	POD 1	1.3 (0.1)	1.5 (0.1)	
	POD 3	1.5 (0.1)	1.3 (0.1)^*^	
	POD 5	1.4 (0.1)^*^	1.3 (0.1)^*^	
	POD 7	1.7 (0.2)	1.3 (0.2)^*^	
Expiratory time (Te), second	Pre (baseline)	2.8 (0.3)	2.2 (0.3)	0.052
	POD 1	1.8 (0.2)^*^	2.7 (0.2)^†^	
	POD 3	2.4 (0.3)	2.1 (0.3)	
	POD 5	2.3 (0.2)	1.9 (0.2)	
	POD 7	2.4 (0.3)	1.8 (0.2)^*^	
Total respiratory cycle time (Ttot), second	Pre (baseline)	4.7 (0.5)	3.7 (0.5)	0.046
	POD 1	3.1 (0.3)^*^	4.2 (0.3)^†^	
	POD 3	3.9 (0.4)	3.5 (0.4)	
	POD 5	3.6 (0.3)^*^	3.2 (0.3)	
	POD 7	4.1 (0.5)^*^	3.1 (0.4)^*^	
Ti/Ttot	Pre (baseline)	0.42 (0.01)	0.40 (0.01)	0.06
	POD 1	0.42 (0.02)	0.36 (0.02)^†^	
	POD 3	0.40 (0.01)	0.39 (0.01)	
	POD 5	0.39 (0.01)^*^	0.42 (0.01)	
	POD 7	0.41 (0.01)	0.42 (0.01)	
Respiratory rate, breaths/minutes	Pre (baseline)	13.2 (1.6)	14.4 (1.5)	0.19
	POD 1	18.3 (1.3)^*^	13.6 (1.2)^†^	
	POD 3	15.3 (1.8)	17.7 (1.7)^*^	
	POD 5	14.8 (2.1)	18.0 (1.9)^*^	
	POD 7	15.5 (2.3)	18.9 (2.1)^*^	

Changes in VAS after laparoscopic gastrectomy are shown in Table 3. There was no significant difference over time at rest and mobilization between the two groups. The VAS at mobilization of the IV-PCA group was significantly higher than that of the PCEA group at T0.

**Table 3:** Changes in visual analog scale after laparoscopic gastrectomy Values are shown as least squares means (standard error). †*p* < 0.05 vs PCEA group. T0, time at leaving operation room; T1, postoperative 6 hours; T2, postoperative 12 hours; T3, postoperative day 1 morning; T4, postoperative day 1 evening; T5, postoperative day 2 morning; T6, postoperative day 2 evening. Abbreviations: IV-PCA, intravenous patient-controlled analgesia; PCEA, patient-controlled epidural analgesia; VAS, visual analog scale.

Visual analog scale	Time	PCEA group (n = 8)	IV-PCA group (n = 8)	*p* value (group×time)
VAS at rest	T0	14 (8)	29 (9)	0.65
	T1	21 (8)	27 (8)	
	T2	25 (9)	26 (9)	
	T3	28 (8)	32 (8)	
	T4	24 (7)	40 (6)	
	T5	30 (6)	37 (6)	
	T6	26 (9)	34 (9)	
VAS at mobilization	T0	9 (9)	41 (9)^†^	0.51
	T1	21 (11)	37 (11)	
	T2	27 (11)	34 (11)	
	T3	34 (9)	45 (8)	
	T4	35 (8)	56 (7)	
	T5	39 (7)	55 (7)	
	T6	33 (9)	48 (9)	

Table 4 shows the recovery of bowel function and hospital stay after laparoscopic gastrectomy. There was no significant difference between the two groups.

**Table 4:** Recovery of bowel function and hospital stay after laparoscopic gastrectomy. Values are represented as median (interquartile range). *Mann-Whitney U test. Abbreviations: IV-PCA, intravenous patient-controlled analgesia; PCEA, patient-controlled epidural analgesia.

Outcomes	PCEA group (n = 8)	IV-PCA group (n = 8)	*p* value^*^
Recovery of bowel function			
Day to first flatus	1.5 (1.0–3.0)	2.0 (2.0–3.0)	0.39
Day to first stool	4.0 (3.5–6.5)	4.0 (3.5–4.0)	0.47
Day to first soft diet intake	3.0 (3.0–3.5)	3.0 (3.0–3.0)	0.95
Hospital stays, days	13.0 (12.0–15.5)	15.0 (14.0–17.0)	0.30

## Discussion

In this study, we compared the effects of PCEA with background infusion and IV-PCA on chest wall mechanics and respiratory timing after laparoscopic gastrectomy. Postoperative diaphragmatic dysfunction, expressed as decreased AB/V_T_, was observed in the IV-PCA group. On the other hand, diaphragmatic dysfunction was partially attenuated in the PCEA group. There were no significant differences in VAS between the two groups. These changes suggested that PCEA with background infusion may confer benefits while providing postoperative analgesia after laparoscopic gastrectomy because of reduced postoperative diaphragmatic dysfunction after surgery.

Diaphragmatic dysfunction has been shown to be responsible for respiratory dysfunction characterized by rapid and shallow breathing after upper abdominal surgery [19]. The cause of diaphragmatic dysfunction after upper abdominal surgery is not fully understood. Several factors, such as postoperative pain and reflex inhibition of the phrenic nerve play an important role in postoperative diaphragmatic dysfunction. Dureuil et al. demonstrated that contractility of the diaphragm is not altered after upper abdominal surgery, and diaphragmatic dysfunction is secondary to reflex inhibition of the phrenic nerve output [6]. Stimulation of visceral nerves, elicited by gallbladder traction or esophageal dilation, markedly decreases phrenic motor neuron output [20,21]. Laparoscopic cholecystectomy impairs diaphragmatic activity and function; however, lower laparoscopic surgery, at the same incision as laparoscopic cholecystectomy, did not cause diaphragmatic dysfunction [22]. In a rat model, upper abdominal incision induced reflex inhibition of phrenic motor activity [23]. These results confirmed that the idea of reflex inhibition of diaphragmatic activity is related to upper abdominal surgery [22,24].

Single-shot epidural analgesia using 0.5% bupivacaine partially improved diaphragmatic dysfunction after upper abdominal surgery [13,14]. These effects were diminished three hours after epidural injection of bupivacaine as regression of epidural analgesia [14]. Epidural injection of bupivacaine improved diaphragmatic dysfunction after upper abdominal surgery accompanied by increases in diaphragmatic electromyogram activity [14]. Pain relief by epidural fentanyl injection could not attenuate postoperative diaphragmatic dysfunction after open cholecystectomy [7]. Intrathecal injection of morphine could not modify the respiratory pattern as characterized by shallow and rapid breathing after upper abdominal surgery [25]. In a rat model, thoracic epidural anesthesia attenuated upper abdominal incision-induced reflex inhibition of phrenic motor activity [23]. These results suggested that blocking the visceral or somatic inhibitory afferents rather than the nociceptive afferent could attenuate postoperative diaphragmatic dysfunction after upper abdominal surgery.

In our study, continuous ropivacaine infusion with PCEA partially attenuated diaphragmatic dysfunction after laparoscopic gastrectomy. On the other hand, pain relief by continuous intravenous administration of fentanyl could not attenuate diaphragmatic dysfunction to some degree after laparoscopic gastrectomy. Our results supported that only blocking the nociceptive afferent could not attenuate postoperative diaphragmatic dysfunction even after laparoscopic upper abdominal surgery, a minimally invasive technique with less postoperative pain. PCEA with background infusion of ropivacaine might attenuate diaphragmatic dysfunction by inhibiting inhibitory reflexes of phrenic activity arising from the upper abdominal compartment. Both PCEA with background infusion and IV-PCA were performed until POD 2. Decreased AB/V_T_ was observed after the removal of PCEA. These results suggested that the effects of epidural analgesia were sustained during epidural local anesthetic administration. However, there were differences in respiratory timing between the two groups on POD 7. In the PCEA group, RR, Ti, and Te returned to preoperative values on POD 7. However, increased RR with shortened Ti and Te were still observed in the IV-PCA group on POD 7. Attenuation of diaphragmatic dysfunction might affect time-course changes in respiratory patterns after upper abdominal surgery. PCEA is favorable for the recovery of respiratory dysfunction after laparoscopic gastrectomy.

The changes in respiratory timing observed in the IV-PCA group on POD 1, including decreased RR and inspiratory duty cycle (Ti/Ttot), seem to represent respiratory depression of opioids [26]. In the PCEA group, although epidural analgesia ameliorated diaphragmatic dysfunction, RR increased without changing the duty cycle. Since we did not measure arterial blood gas and V_T_, we could not clarify the exact mechanisms of increased RR. V_T_ might be decreased compared to preoperative levels because epidural analgesia might paralyze lower intercostal muscles leading to reduced V_T_. It might represent a compensatory response to reduced V_T_ after laparoscopic gastrectomy and changes in chest wall conformation and resting length, and a shift of the workload of breathing from the rib cage to the diaphragm caused by epidural analgesia [27]. Inspiratory muscle dysfunction causes tachypnea with increasing inspiratory duty cycle in patients with chronic obstructive pulmonary disease [28].

Some limitations should be noted. First, the anesthesiologists, surgeons, nurses, and patients could not be masked from the analgesia method because of the study design. However, chest wall mechanics and respiratory timing are objective evaluation methods, so observer bias is unlikely. Second, the small sample size limits the interpretation of the results of the present study. Especially the result of VAS, the recovery of bowel function, and the duration of hospital stay, the analysis lacked sufficient statistical power. Although power analysis should be performed before the start of the study, we sought to enroll as many eligible patients as possible during the study period approved by the Research Review Board at our hospital. However, the chest wall mechanics and respiratory timing results were statistically significant even at this sample size. Third, there are significant differences in age and BMI between the two groups despite randomization. Age was significantly older in the IV-PCA group, while BMI was significantly higher in the PCEA group. Older age may mean that it can take longer to recover diaphragmatic function after surgery, leading to better AB/V_T_ in the PCEA group than in the IV-PCA group. On the other hand, higher BMI is more likely to decrease postoperative vital capacity, which might lead to better AB/V_T_ in the IV-PCA group than in the PCEA group. These differences were considered to happen by chance, probably because of the small sample size, and the present analyses were performed without any correction. In a sensitivity analysis adding age and BMI as covariates to the relevant model, the result did not change materially (data not shown). Finally, our study protocol did not include rules regarding the administration of muscle relaxants; it might affect the level of intraoperative muscle relaxation and subsequently diaphragm fatigue. However, there was no significant difference between the two groups in the total dose of muscle relaxants per weight during surgery.

## Conclusions

In our study, continuous ropivacaine infusion with PCEA partially attenuated diaphragmatic dysfunction after laparoscopic gastrectomy. Meanwhile, pain relief by continuous intravenous administration of fentanyl could not attenuate diaphragmatic dysfunction. This suggests that PCEA might ameliorate postoperative diaphragmatic dysfunction after laparoscopic gastrectomy. Further investigation with a larger sample size is needed to elucidate whether PCEA would be superior to IV-PCA in reducing pulmonary complications and pain scores after laparoscopic abdominal surgery.
